# JTrack: A Digital Biomarker Platform for Remote Monitoring of Daily-Life Behaviour in Health and Disease

**DOI:** 10.3389/fpubh.2021.763621

**Published:** 2021-11-19

**Authors:** Mehran Sahandi Far, Michael Stolz, Jona M. Fischer, Simon B. Eickhoff, Juergen Dukart

**Affiliations:** ^1^Research Centre Jülich, Institute of Neuroscience and Medicine, Brain and Behaviour (INM-7), Jülich, Germany; ^2^Medical Faculty, Institute of Systems Neuroscience, Heinrich Heine University Düsseldorf, Düsseldorf, Germany

**Keywords:** mobile toolkit, mobile sensing, remote monitoring, health science, biomarkers

## Abstract

Health-related data being collected by smartphones offer a promising complementary approach to in-clinic assessments. Despite recent contributions, the trade-off between privacy, optimization, stability and research-grade data quality is not well met by existing platforms. Here we introduce the JTrack platform as a secure, reliable and extendable open-source solution for remote monitoring in daily-life and digital-phenotyping. JTrack is an open-source (released under open-source Apache 2.0 licenses) platform for remote assessment of digital biomarkers (DB) in neurological, psychiatric and other indications. JTrack is developed and maintained to comply with security, privacy and the General Data Protection Regulation (GDPR) requirements. A wide range of anonymized measurements from motion-sensors, social and physical activities and geolocation information can be collected in either active or passive modes by using JTrack Android-based smartphone application. JTrack also provides an online study management dashboard to monitor data collection across studies. To facilitate scaling, reproducibility, data management and sharing we integrated DataLad as a data management infrastructure. Smartphone-based Digital Biomarker data may provide valuable insight into daily-life behaviour in health and disease. As illustrated using sample data, JTrack provides as an easy and reliable open-source solution for collection of such information.

## Introduction

Neurological and psychiatric diseases typically present with symptoms that are complex, atypical, fluctuant in disease progression, and display high variability between patients (1). Current diagnostic and efficacy evaluation methods often rely on in-clinic visits and subjective evaluation by patients, caregivers or clinicians. In-clinic evaluation methods are often costly, time-consuming and limited in their quality and quantity of observations (2). In addition, they are often prone to high inter- and intra-rater variability (3). The aforementioned drawbacks of traditional diagnosis methods may affect the diagnostic process especially in the early stage of the disease where there is a lag between the onset of the pathological process and the onset of symptoms (4).

Psychiatric and neurological diseases are typically long-term illnesses that cause significant fluctuations in symptoms over time. Therefore, recall and reporting biases are the key difficulties in evaluating respective diseases in episodic in-clinic visits. Remote monitoring of patients in their everyday-life using sensor-based at smart technologies is rapidly evolving and may assist clinicians in facilitating early diagnosis and evaluating and adjusting interventions. There has been an evolving interest in using newly emerged smart sensor technologies for monitoring of patients (5–10).

Modern smartphones and wearables are equipped with various sensors including motion (i.e., acceleration, gyroscope), location [i.e., Global Positioning System (GPS)], environment (i.e., barometer, temperature, light) and health sensors (i.e., heart rate) (11, 12). This rich combination of sensors along with their ability to collect ecological momentary assessments (EMA), and information about social interaction (i.e., social media, messaging and phone calls) have made smartphones a potential alternative to in-clinic evaluation for various types of assessments (13, 14). Such health-related information being collected in clinical trials are often referred to as digital-biomarkers (DB) (15). DBs can provide objective, ecologically valid, and invaluable information for better understanding of specific diseases. In addition, DBs enable frequent assessments from larger target populations over longer periods of time and may thus provide detailed insight into inter- and intra-individual disease variability in daily-life (16).

Several contributions enabling the use of smartphones as an assessment tool have been recently introduced. The first set are commercial devices such as Fitbit^1^, Garmin^2^, Apple^3^ and Samsung^4^ devices. The main focus of these applications is to provide feedback on the daily activity of users by visualizing and showing notification regarding their heart rate, number of steps and kind of activity. However, most of these devices provide limited access to the raw data and do not support high-frequency data collection. A second type are applications and platforms developed by researchers such as AWARE (17), RADAR-base (18), Beiwe (19), mCerebrum (20), mPower (21) and many others. The main focus of these mostly open-source platforms is to enable data collection for research applications as well as to facilitate data sharing and reproducibility. Yet, these software packages are often limited by an often narrow focus to some specific clinical indications or with respect to privacy aspects. Also, these once in a while updated platforms make some of them unstable for the rapidly growing smartphone ecosystem.

Whilst there are several platforms that are able to collect context-driven data, the trade-off between privacy, optimization, stability and research-grade data quality is not well met yet. Thus, we aim to fill this gap by introducing the JTrack platform. JTrack was developed as an Android-based application for smartphones and an online server-side dashboard. JTrack application comprises the following main categories of components: sensor data, location data, Human Activity Recognition (HAR), and smartphone and application usage monitoring. Each component has the option to be used for active (with user interaction) and passive (without user interaction) monitoring. The dashboard side is an online platform to create and manage studies, integrating DataLad (22) infrastructures to facilitate management and sharing of collected data. JTrack is a modular open-source with a high level of optimization, security and privacy making JTrack a practical solution for clinicians and researchers to collect, manage, and share digital biomarker data.

## Methods

### General Description

**Figure 1:**
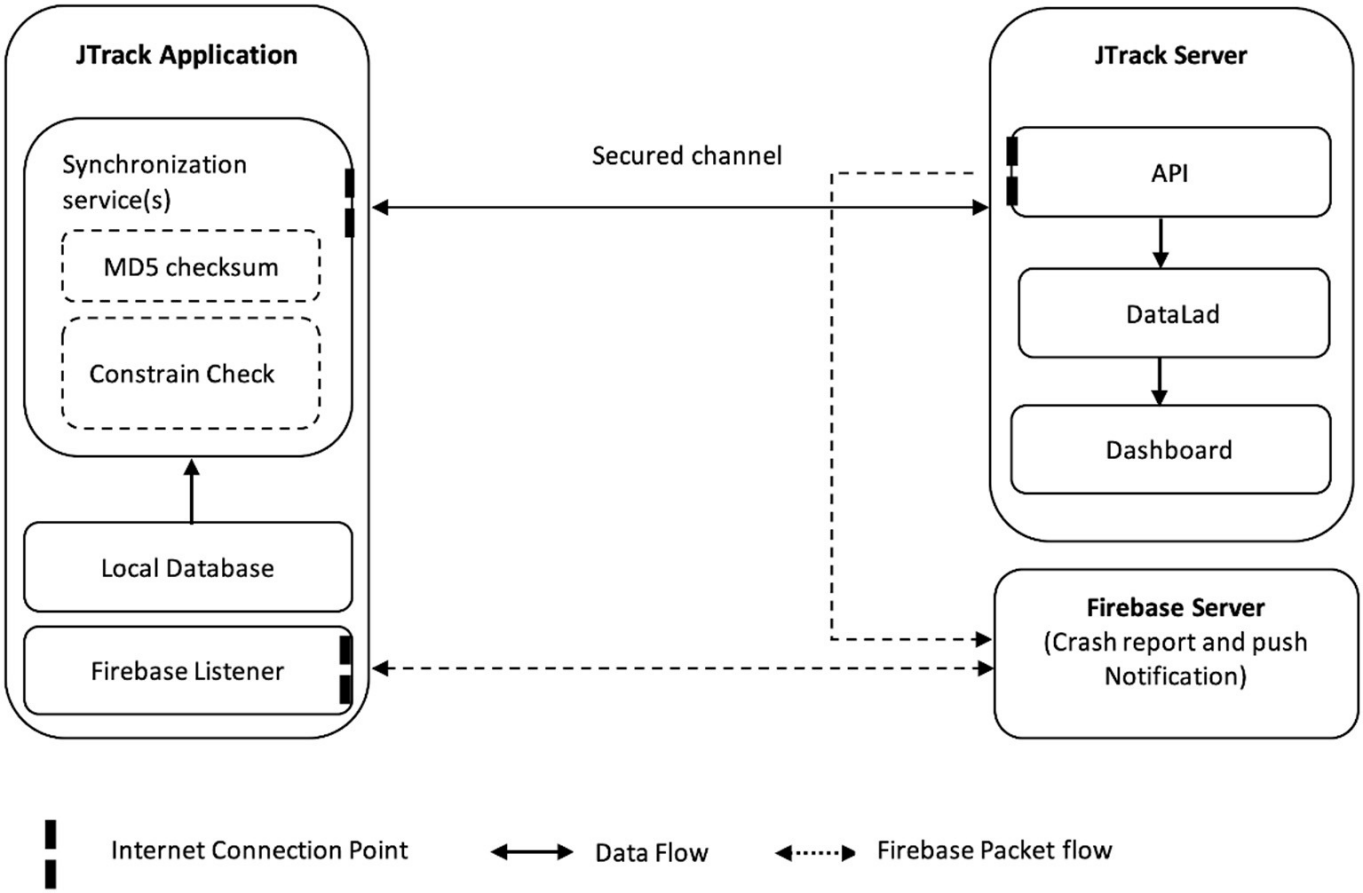
JTrack Platform overview.

**Figure 2:**
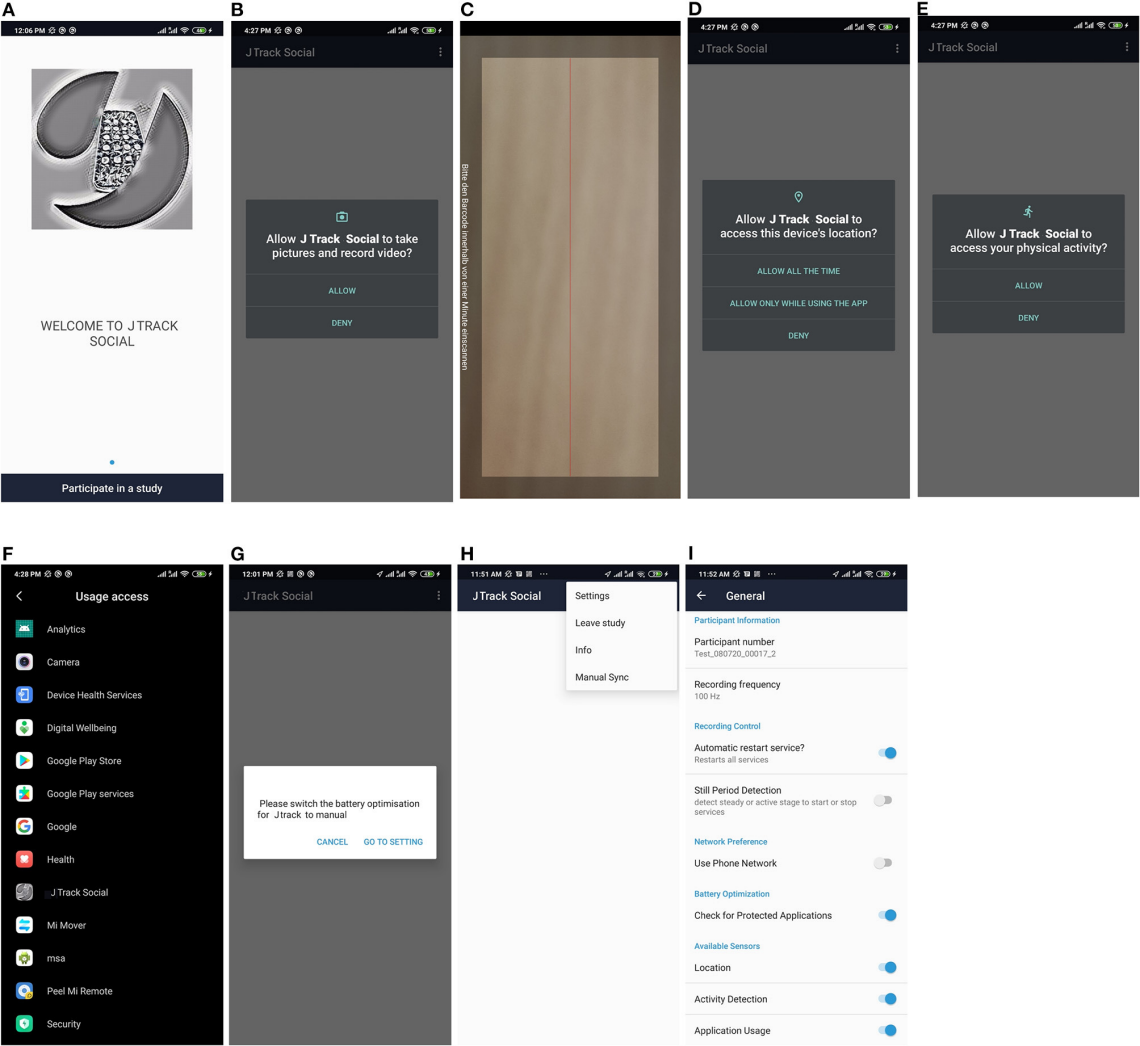
JTrack Application Environment. **(A)** opening page of the application, **(B)** requesting for camera permission, **(C)** QR-Code scanner, **(D)** requesting for location permission, **(E)** requesting for an activity detection permission, **(F)** referring to ask for usage permission which is used for application usage module, **(G)** detecting a custom optimization and asking for disabling it, **(H)** the main menu of the application, where users may access the administration menu, leave a study, get information about the application and do manual synchronization, **(I)** administration menu, here we have access to the main setting of application, the information provided here is for further administration from study owners and most of the information is catch from sever during Installation.

**Figure 3:**
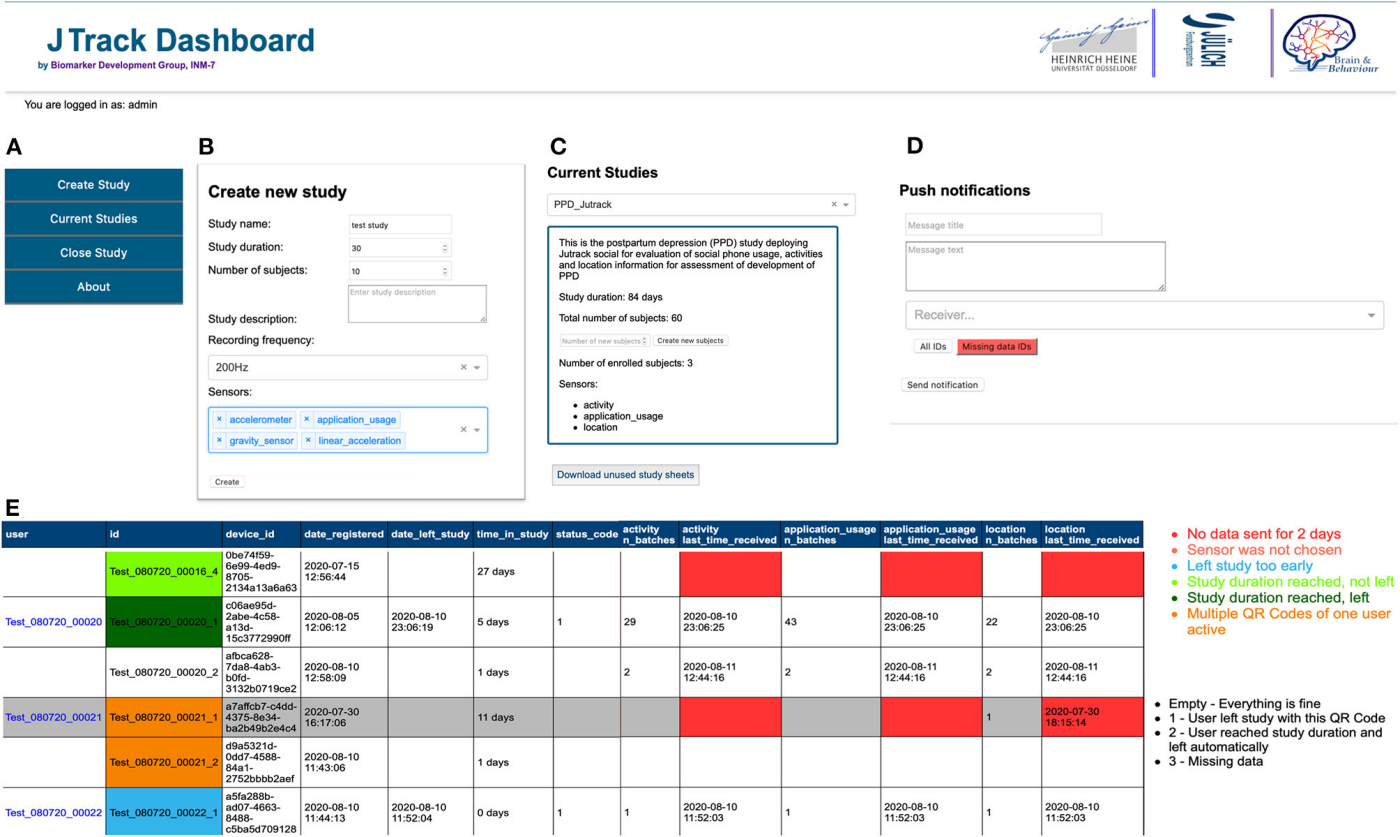
JTrack Dashboard Environment. **(A)** main menu, **(B)** here a new study can be created by specifying its details such as duration of the study, number of users, and list of data categories to be collected, **(C)** currently ongoing studies and details of the selected study can be found here. Also, the generated QR-Code can be downloaded here, **(D)** to accomplish more interaction with users participated in a particular study, a message as a push notification can be sent to a target user(s), **(E)** details of received data, date of registration, date left, duration within in study and quality control by color-labeling for sent data for users in a selected study can be monitored here.

Here we introduce the main components of the JTrack platform (Figure 1) comprised of the JTrack app (Figure 2) and an online dashboard interface (Figure 3). The smartphone application JTrack was developed for smartphones with the Android operation system (OS). The reason for selecting Android was a wider range of users^5^ (73%) and fewer restrictions which were necessary for technical aspects of application development.

JTrack enables passive 7/24 data collection running in the background. Active data collection is enabled through simple interaction (i.e., start and stop recording, i.e., before and after execution of a specific task). All collected data are recorded locally in the application and then synchronized on a periodic basis (i.e., connection to the Internet, have enough battery charge). All local data are deleted from the phone storage upon successful data transfer. To minimize the risk of data loss, we implemented auto-start functionality (without user interaction) to resist unwanted application crashes or operating system reboots, and all the crashes are reported via the Firebase dashboard^6^.

On the server side, the JTrack dashboard was developed as an online web-application where study owners can create and manage studies. The dashboard consists of a front-end interface and a back-end API which is integrated with DataLad (22) as a data-management tool. The dashboard provides an overview of received data including sanity checks such as MD5 for received data, and embedded validity checking methods.

### QR-Code Authentication

To provide a convenient and secure way of activation we implemented a QR-code method. The QR-code for each subject is generated as a pdf file from the dashboard. Each QR-code contains all the necessary information such as user ID, Study ID, and address of the target server or an optional authentication method (e.g., OAuth2). To join a study, the one-time QR-code needs to be scanned using the QR-scanner embedded in the JTrack app. Additional backup QR-codes are provided for scenarios in which users may want to leave and re-join or need to switch their device.

### Location Service

Location service provides an update on visited location data such as longitude, latitude, and altitude. This service operates as a part of the passive recording. The location data can be inquired based on pre-defined periods (i.e., 10 min). To ensure anonymization, for each user, a random value is generated during activation on the phone, which shifts the latitude to a random place on the globe. In addition, all recorded coordinates are rotated using a randomly generated fixed degree around this initial coordinate to ensure that even if the true installation location is known no other coordinates can be derived. These values remain on the phone throughout the study and are deleted upon deinstallation of the app. To keep a high accuracy, each data point is first transferred from WGS-84 to Cartesian coordinates. After the transformation using the generated value, the coordinates are transferred back to their native space. Since this transformation occurs before actual recording, all the collected data is relative and cannot be used to recover the user's actual location. Furthermore, we used a fused method that provides more accurate data (median accuracy of 14 meters) by combining GPS and network information.

### Human Activation Recognition

Inertial Measurement Unit (IMU) sensors of smartphones or wearables can be used to differentiate between human activities. Several studies described reliable algorithms for HAR (23, 24). Nowadays, these algorithms are routinely deployed in commercial devices, as well as in a wide range of research areas from medicine to military. JTrack uses the Google Play Activity Recognition Service^7^ for HAR, which recognizes up to six types of activity (walking, running, still, on bicycle, on vehicle or tilting). JTrack can record the detected activity and the assigned certainty with a pre-specified frequency of 5 min. The HAR module is computationally lightweight, optimized and does not require direct access to raw sensor data.

### Application Usage Statistic

JTrack can collect the statistic of user's interactions with the smartphone. This data includes the name of the application and the amount of time it is used in the foreground since the previous midnight. Phone calls and SMS are treated as applications with same usage statistics being collected as above. No content of the applications, messages or phone calls (including phone numbers) is collected at any stage.

### Sensors

Various sensors are embedded in any modern smartphones which are classified as hardware implementation (i.e., accelerometer, gyroscope, barometer) or software implementation (i.e., rotation sensor). JTrack enables collection of data from most of the available sensors depending on the device model and version of Android. Among these, accelerometer, gyroscope and gravity sensors are the most important sensors for researchers focusing on motion analysis (6, 25–29). As a default, JTrack provides recording of accelerometer and gyroscope data in the passive collection mode. Other sensors can be added upon the researcher's choice by using the provided template module which requires minimal coding effort. For each sensor, sampling frequency in Hz can be adjusted using the dashboard when creating a new study.

### Dashboard

When creating a new study in the dashboard, all aspects of a study such as study name, duration, number of subjects, recording frequency, and categories of data to be collected can be customized. After creating a study, the dashboard will generate QR-codes which are used for enrolment into the study. The dashboard also provides management tools on an ongoing study producing information such as a number and time of received data for each sensor/modality and status (i.e., active, not active) of each participant in a particular study. We also implemented several quality controls including highlighting of missing data.

Furthermore, to assist study managers to establish further interaction with participants, we embedded a messaging method in the dashboard which allows to send a push notification directly to the participants' phone, either by selecting specific subject numbers or all participants within a particular study. Layered design (backend, frontend and data management layer) also makes the Dashboard flexible and extendable for further interaction and integration with third-party applications.

### Performance, Security and Privacy

At all stages of the development, attention was paid to security and privacy as a main priority. In this context, we designed the JTrack platform to comply with GDPR and Google Developer Policies^8^. No sensitive data such as name, phone number, phone contacts or actual location are recorded at any stage. All the collected data transferred via Hypertext Transfer Protocol Secure (HTTPS) protocol and checked for any inconsistency using MD5 sanity checksum.

Concerning patient privacy, all users using JTrack are provided with clear information on what is been recorded and why. Permission requests for each module need to be approved during installation and activation. All participants may also stop and leave a study at any time directly from the app. Also, remote configuration and one-step recording allow clinicians to gain optimum control over the collected data without the need to collect any identifying information.

To reduce battery and memory usage, we provided several built-in optimizations such as:

- Detecting still period of the phone to pause recordings.
- Delete locally stored data right after synchronization with the server.
- Scheduled synchronization based on predefined criteria such as access to a Wi-Fi connection.
- Detect and provide a possibility to bypass performance optimizations (i.e., battery and memory) policies of phone manufacturers introduced on-top of the Android OS.

To reduce data loss due to crashes or reboots, automatic re-starting is implemented alongside with Firebase integration to obtain performance and crash reports. Information about phone manufacturer, model, and OS version are among the optional recorded data (not active by default), which can be used to analyse and handle cross-sensor variability.

### Pilot Study

In a pilot experiment, we collected in a cohort of healthy volunteers (*N* = 21, age: 26.1 ± 6.9, 7 female) for 2 weeks on a daily basis passively recorded data for application usage, location and activity recognition aside with self-reported estimates for these parameters [for application usage: time spent (in minutes) with the phone: total, social media and messenger]; for location and activity: walking/running distance (in meters). To test for associations between passively recorded and self-reported measures, we computed Pearson correlations across all subjects and time-points to compare both types of measures (i.e., merging location and activity recognition co compute distance covered by foot).

## Results

**Figure 4:**
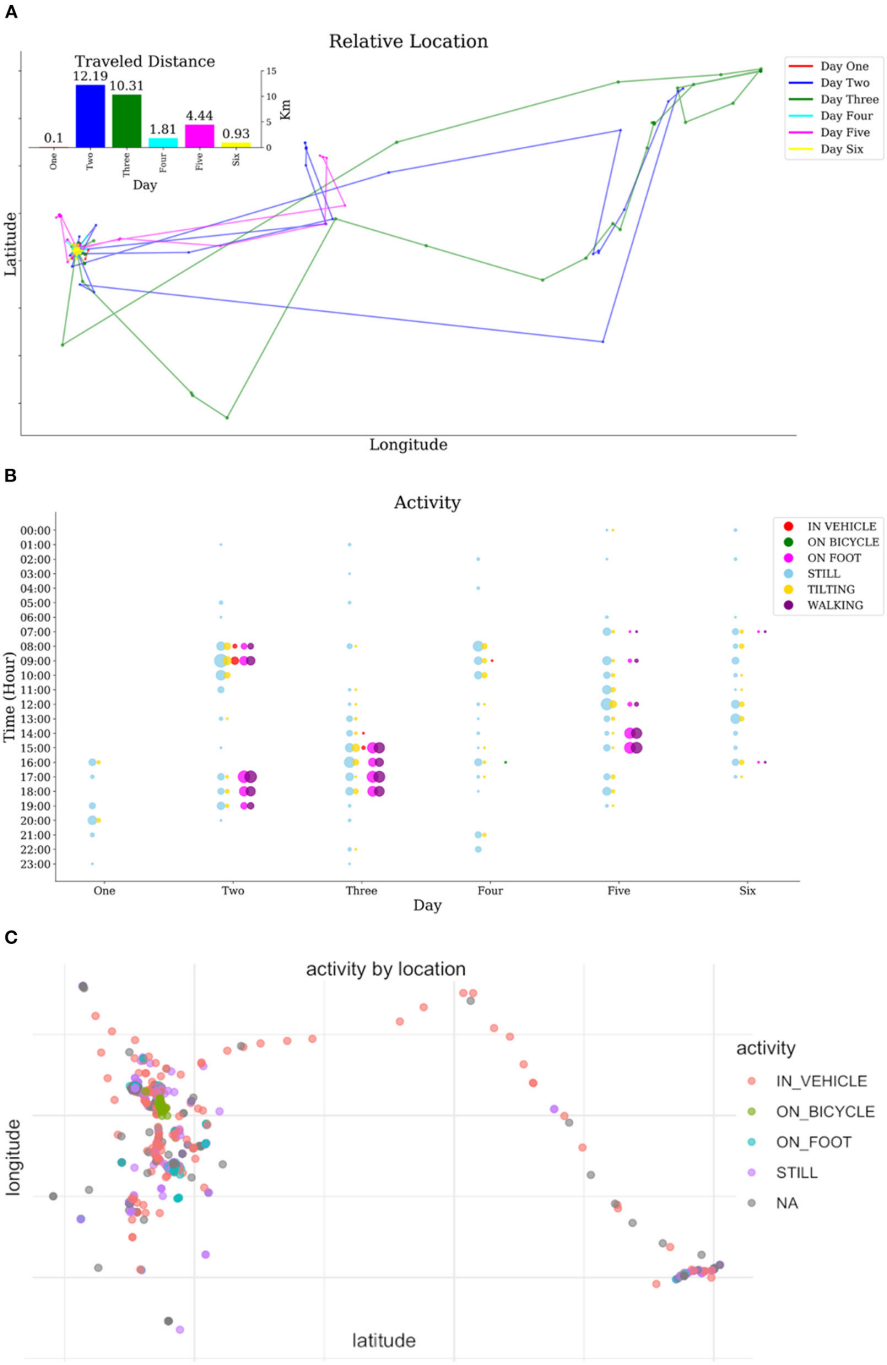
Data sample for activity and location modules. **(A)** traveled distance and relative geolocation information for different days, **(B)** distribution of different physical activates during a different time of different days, **(C)** type of activity data combined with geolocation information.

**Figure 5:**
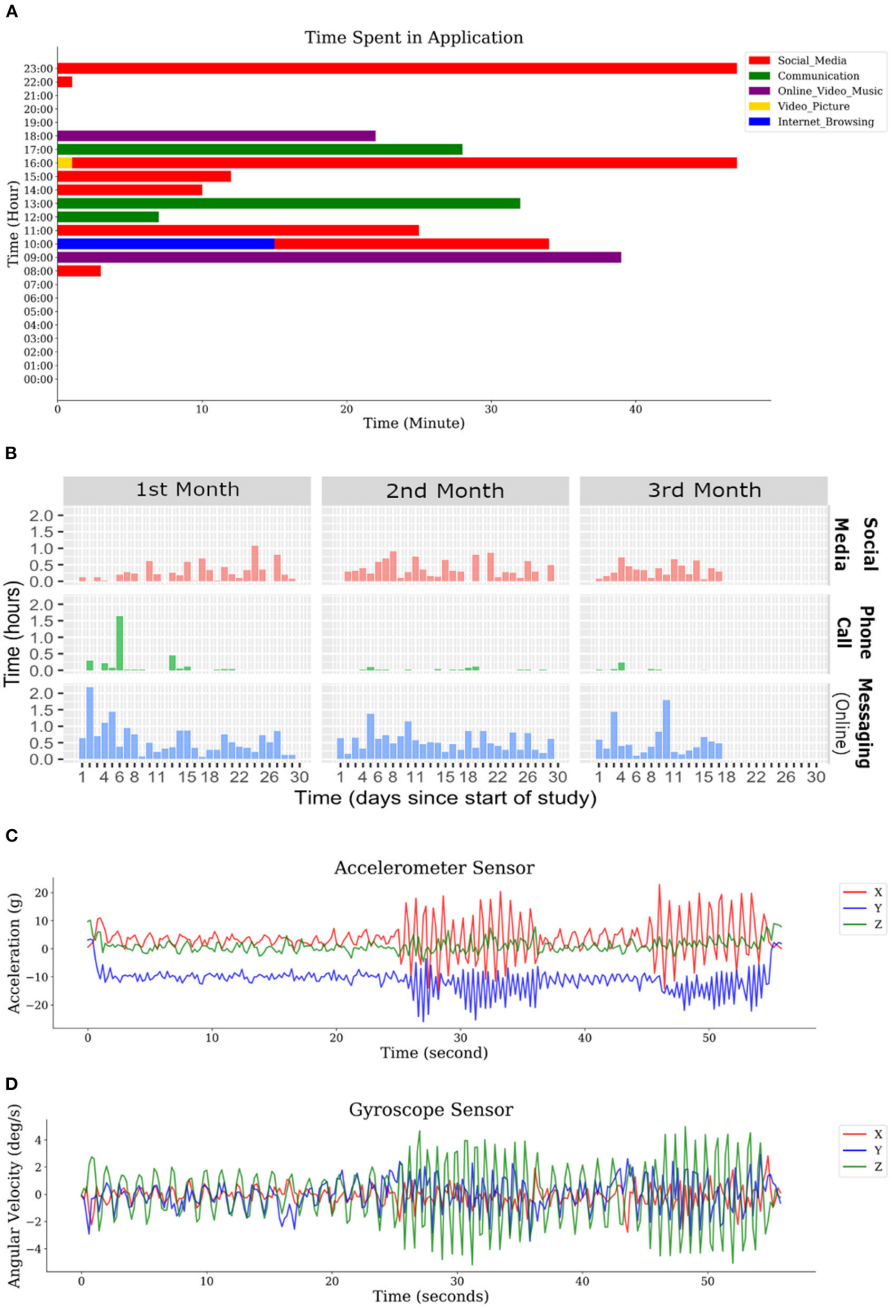
Data sample for application usage module and sensors. **(A)** amount of time spent in different application types for a day, **(B)** distribution of different type of application usage during a study. **(C)** raw data recorded from the acceleration sensor, **(D)** raw data recorded from the Gyroscope sensor.

To illustrate the utility of the JTrack application sample data were collected in the beta testing phase. Figures 4, 5 display such sample data collected for a single subject for different modalities. We provided sample data for each modality (i.e., location data, activity recognition, application usage and raw sensors data) also we further show a possible combination of the recorded data (i.e., location data with activity recognition data) in Figure 4C. Other combinations such as time and location (e.g., extracting amount of time spend outside of common residual place), location and application usage (e.g., extracting pattern of social interaction and applications used in-home condition) and activity and raw sensor data (e.g., extracting driving behaviour) are among the possible ways of making inference. Figure 5B also shows the daily phone and application usage (i.e., social media, phone calls, and online messaging platform) for a pilot participant.

**Figure 6:**
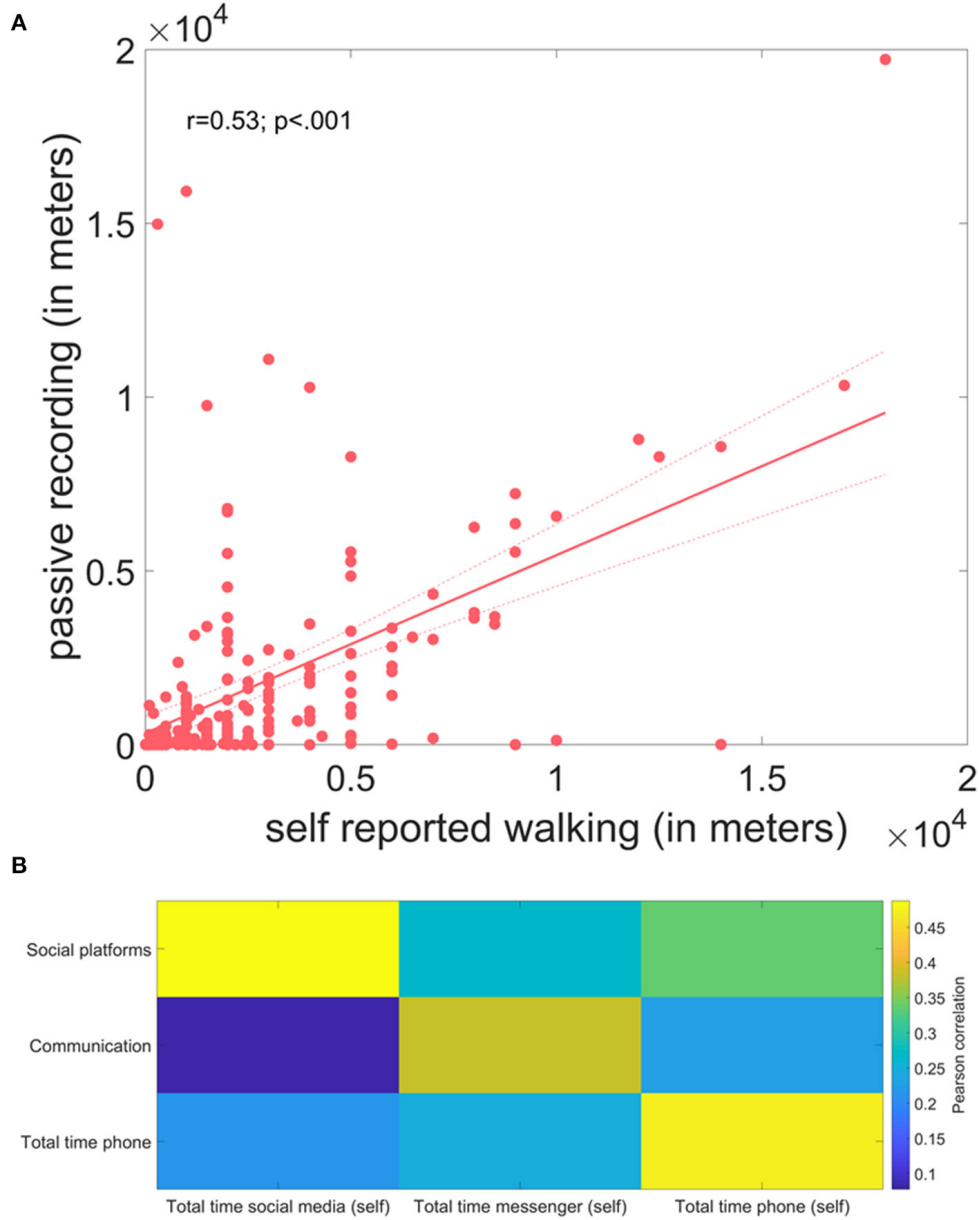
Results of the pilot study comparing self-reported and passively recorded measures of daily behaviour. **(A)** Correlation plot between self-reported distance walking/running and distance estimates derived from fusion of location and activity recognition data. **(B)** Correlation matrix comparing self-reported measures of different smartphone usages and estimates derived from passively recorded app usage information.

In a pilot experiment, we further tested for associations between these passively recorded measures and self-reported estimates of specific behaviours. The self-reported distance covered by walking/running per day significantly correlated (*r* = 0.53; *p* < 001) with the information derived from passive monitoring (Figure 6A). Similarly, the time spent with the phone in total, communication and social platform significantly correlated with the passively-recorded estimates of respective phone usage measures (*r* = 0.38–0.49; all *p* < 0.001) (Figure 6B).

### Comparison to Other Platforms

Table 1 shows how the JTrack platform compares to other similar and related platforms in terms of some key features such as security and privacy, activation, management and also stability. AWARE (17) is a platform for remote assessment of a wide range of phone sensors, activity and self-reported data. AWARE also supports additional plugins for external sensors and new data. However, this ability also requires a further declaration of permissions which limits control over privacy. mCerebrum (20) is another platform for remote assessment supporting a wide range of high-frequency sensors with a focus on energy-optimization. However, this platform has not been updated in while (latest update is May 2018 in their GitHub repository), questioning its performance on new versions of Android-OS. Beiwe (19) is the next platform supporting remote monitoring and DBs assessments which has a flexible study portal, modeling and data analysis tools. Nevertheless, this platform does not have local data storage and makes use of Amazon Web Services (AWS) cloud computing infrastructure. Such public cloud-based solutions are often more cost effective and convenient to use since as they simplify the build and maintenance process (this is particularly evident when the number of users and the data collected are small to medium sized), yet they may also raise data privacy questions and require additional deployment procedures. Another drawback of this platform is the collection of identifiable data such as phone number, media access control (MAC) address of WIFI and Bluetooth devices. RADAR-base (18) is the last open-source platform in the list. It has a well-organized structure which is using Confluent and Apache Kafka services and flexible study portal. Nevertheless, the deployment and adaptation of this platform require heavy configuration. Concerning the convenient registration, it requires text-based registration. Location data being collected in background is considered as a big concern in terms of privacy which is also frequently regulated by Google Developer Policies^9^ and restricted by recent updates in Android OS. Among all the compared platforms only RADAR-base provides relative location. There are several alternative variants of these platforms that may strengthen some of the basic capabilities, such as Health Outcomes through Positive Engagement and Self-Empowerment (HOPES) which is based on the Beiwe platform (30) and AWARE-Light which is based on the aware framework. While these additional enhancements may address some of the shortcomings of the specific underlying platform, here we only focused on the comparisons to the core versions.

**Table 1:** Comparison of existing frameworks with JTrack.

**Framework**	**Location Anonymization**	**Official app Stores**	**Data Management**	**Remote Configuration**	**Activation**	**Customized OS Detection**
AWARE (17)	NO	NO	SQL	YES	Text-based	NO
RADAR-base (18)	YES	YES	MongoDB	YES	QR-Code	NO
Beiwe (19)	NO	YES	PostgreSQL	NO	Text-based	NO
mCerebrum (20)	NO	NO	MySQL	NO	Text-based	NO
JTrack (this study)	YES	YES	DataLad	YES	QR-Code	YES

Easy one-step registration and authentication via QR-Code, as well as remote configuration, make JTrack more practical in both the usage and management aspects. Battery and memory optimizations offered by Android OS or phone manufacturers can affect the stability and consistency of data collected, JTrack provides built-in detection and circumvention methods for better stability that are not provided by comparator platforms at this level.

## Discussion

We developed JTrack as an open-source, smartphone-based platform for digital phenotyping. JTrack consists of a smartphone application and an online dashboard enabling remote data collection and study management. JTrack provides a flexible and modular environment for collection of various types of sensor and smartphone usage data with particular attention being paid to patient privacy as well as compliance with GDPR regulations.

From the functionality perspective, most of the solutions described above were developed with the focus on specific applications, i.e., a specific disease [i.e., RADAR-base (18) and Beiwe (19)]. Their application is therefore limited to the respective primary context. In contrast, some other platforms were developed to collect as much information as possible with little attention to data privacy [i.e., AWARE (17)]. Such frameworks violate GDPR and Google Play Store policies limiting their deployment for many clinical applications. JTrack aims to fill this gap by providing a customizable platform that can be deployed across different indications whilst paying large attention to privacy and security policies. JTrack aims to comply with GDPR regulations as well as with the Google Play Store policies. It only requires minimal access to the device information and avoids collection of identifiable or sensitive data.

Developing an application for smartphones always requires dealing with variation in devices (e.g., manufacture, screen size, available sensors) as well as the variation of operation systems (OS) versions. Different manufacturers may add further OS optimizations such as limiting background processes. This may cause inconsistencies in performance of monitoring applications. We introduced several layers to detect, report and prevent the side effects of these variations. JTrack is actively maintained and covers up to 84.9% of Android smartphones [Minimum Software Development Kit (SDK) 23] dealing currently with Android optimizations from eight main Android smartphone manufactures. Although the JTrack platform is now only available for the Android environment, which may introduce selection bias and limit participants to having an Android smartphone, an iOS version of JTrack is currently in development, with similar capabilities and will be made publicly available in the same GitHub repository and under the same open-source license.

Potential applications for JTrack include but are not limited to monitoring of motion information in diseases associated with alterations of gait and other motor functions affecting phone use. Similarly, the ability to track phone usage allows for monitoring of different types of behaviour, i.e., phone-based social interaction. As such, JTrack may be useful to track such behaviours in healthy participants as well its alterations by specific disorders.

Finally, to facilitate the reusability, JTrack is released under open-source Apache 2.0 licenses. All modules including online-management dashboard can be adopted and extended. It has been designed with modular structure to enable flexibility and customization to support new data and sensors.

Variations in device model, Android version, network quality, and other technical features may have negative effects on the performance of JTrack. Despite the effort to minimize crashes and data loss, there is no guarantee for such. During the development process, we used different third-party services (e.g., Google Play Service), any change or deprecation in these services, or Android policies may also affect the functionalities of JTrack partly or as a whole. Lastly, JTrack was designed and tested for smartphones. It may be used on other devices such as wearables (i.e., smartwatches) or tablets but further tests should be considered beforehand.

JTrack is an active and open-source project which is continuously maintained. We consistently improve and add new features to the platform. The features described here are part of the v1 release. Newer versions may differ and include additional functionalities at the time this article is published.

## Data Availability Statement

For the most updated and previous versions please visit the public repository accessible at https://github.com/Biomarker-Development-at-INM7. The raw data supporting the conclusions of this article will be made available by the authors, without undue reservation.

## Ethics Statement

The studies involving human participants were reviewed and approved by Ethikkommission an der Medizinischen Fakultät, Heinrich-Heine-Universität Düsseldorf Moorenstraße 5 40225 Düsseldorf. The patients/participants provided their written informed consent to participate in this study.

## Author Contributions

MSF wrote the android-based application and performed all analysis. MSF wrote the manuscript with support of JD. MS and JF wrote the dashboard online application. JD designed the overall study. SE contributed to the overall design of the study. All authors contributed to the article and approved the submitted version.

## Funding

This study was supported by the Human Brain Project, funded from the European Union's Horizon 2020 Framework Programme for Research and Innovation under the Specific Grant Agreement No. 785907 (Human Brain Project SGA2).

## Conflict of Interest

JD is a former employee and received consultancy fees on another topic from F. Hoffmann-La Roche AG. The remaining authors declare that the research was conducted in the absence of any commercial or financial relationships that could be construed as a potential conflict of interest.

## Publisher's Note

All claims expressed in this article are solely those of the authors and do not necessarily represent those of their affiliated organizations, or those of the publisher, the editors and the reviewers. Any product that may be evaluated in this article, or claim that may be made by its manufacturer, is not guaranteed or endorsed by the publisher.

## Footnotes

^1^https://www.fitbit.com/global/us/home (accessed November 23, 2020).

^2^https://www.garmin.com/en-US/ (accessed November 23, 2020).

^3^https://www.apple.com/ (accessed November 23, 2020).

^4^https://www.samsung.com/us/ (accessed November 23, 2020).

^5^https://gs.statcounter.com/os-market-share/mobile/worldwide (accessed August 19, 2020).

^6^https://firebase.google.com (accessed November 23, 2020).

^7^https://developers.google.com/location-context/activity-recognition (accessed June 23, 2020).

^8^https://developer.android.com/distribute/play-policies (accessed December 2, 2020).

^9^https://developer.android.com/distribute/play-policies (accessed December 2, 2020).

## References

[1] Lyketsos CG Kozauer N Rabins PV. Psychiatric manifestations of neurologic disease: where are we headed? Dialogues Clin Neurosci. (2007) 9:111–24. 10.31887/DCNS.2007.9.2/clyketsos 17726911 PMC2687521

[2] Zhan A Little MA Harris DA Abiola SO Dorsey ER Saria S . High frequency remote monitoring of Parkinson's disease via smartphone: platform overview and medication response detection. arXiv Preprint arXiv:1601.00960. (2016).

[3] Rovini E Maremmani C Cavallo F. How wearable sensors can support parkinson's disease diagnosis and treatment: a systematic review. Front Neurosci. (2017) 11:555. 10.3389/fnins.2017.00555 29056899 PMC5635326

[4] Balogh EP Miller BT Ball JR Committee on Diagnostic Error in Health Care, Board on Health Care Services, Institute of Medicine . Improving Diagnosis in Health Care. Washington, DC: National Academies Press (2015). 26803862

[5] Rahlf AL Petersen E Rehwinkel D Zech A Hamacher D. Validity and reliability of an inertial sensor-based knee proprioception test in younger vs. older adults. Front Sports Act Living. (2019) 1. 10.3389/fspor.2019.00027 33344951 PMC7739624

[6] Orlowski K Eckardt F Herold F Aye N Edelmann-Nusser J Witte K. Examination of the reliability of an inertial sensor-based gait analysis system. Biomed Tech. (2017) 62:615–622. 10.1515/bmt-2016-0067 28099115

[7] Hasegawa N Shah VV Carlson-Kuhta P Nutt JG Horak FB Mancini M. How to select balance measures sensitive to Parkinson's disease from body-worn inertial sensors-separating the trees from the forest. Sensors. (2019) 19. 10.3390/s19153320 PMC6696209 31357742

[8] Skodda S Grönheit W Mancinelli N Schlegel U. Progression of voice and speech impairment in the course of Parkinson's disease: a longitudinal study. Parkinsons Dis. (2013) 2013:389195. 10.1155/2013/389195 24386590 PMC3872441

[9] Cancela J Pastorino M Arredondo MT Nikita KS Villagra F Pastor MA. Feasibility study of a wearable system based on a wireless body area network for gait assessment in Parkinson's disease patients. Sensors. (2014) 14:4618–33. 10.3390/s140304618 24608005 PMC4003960

[10] Serra-Añó P Pedrero-Sánchez JF Hurtado-Abellán J Inglés M Espí-López GV López-Pascual J. Mobility assessment in people with Alzheimer disease using smartphone sensors. J Neuroeng Rehabil. (2019) 16:103. 10.1186/s12984-019-0576-y 31412893 PMC6694667

[11] Bandodkar AJ Wang J. Non-invasive wearable electrochemical sensors: a review. Trends Biotechnol. (2014) 32:363–71. 10.1016/j.tibtech.2014.04.005 24853270

[12] Khan Y Ostfeld AE Lochner CM Pierre A Arias AC. Monitoring of vital signs with flexible and wearable medical devices. Adv Mater Weinheim. (2016) 28:4373–95. 10.1002/adma.201504366 26867696

[13] Kraft R Schlee W Stach M Reichert M Langguth B Baumeister H . Combining mobile crowdsensing and ecological momentary assessments in the healthcare domain. Front Neurosci. (2020) 14:164. 10.3389/fnins.2020.00164 32184708 PMC7058696

[14] Harari GM Lane ND Wang R Crosier BS Campbell AT Gosling SD. Using smartphones to collect behavioural data in psychological science: opportunities, practical considerations, and challenges. Perspect Psychol Sci. (2016) 11:838–54. 10.1177/1745691616650285 27899727 PMC5572675

[15] Insel TR. Digital phenotyping: technology for a new science of behaviour. JAMA. (2017) 318:1215–6. 10.1001/jama.2017.11295 28973224

[16] Swan M. Crowdsourced health research studies: an important emerging complement to clinical trials in the public health research ecosystem. J Med Internet Res. (2012) 14:e46. 10.2196/jmir.1988 22397809 PMC3376509

[17] Ferreira D Kostakos V Dey AK. AWARE: mobile context instrumentation framework. Front ICT. (2015) 2:1–9. 10.3389/fict.2015.00006

[18] Ranjan Y Rashid Z Stewart C Kerz M Begale M Verbeeck D . RADAR-base: an open source mHealth platform for collecting, monitoring and analyzing data using sensors, wearables, and mobile devices. JMIR Mhealth Uhealth. (2018) 7:e11734. 10.2196/preprints.11734 31373275 PMC6694732

[19] Torous J Kiang MV Lorme J Onnela J-P. New tools for new research in psychiatry: a scalable and customizable platform to empower data driven smartphone research. JMIR Ment Health. (2016) 3:e16. 10.2196/mental.5165 27150677 PMC4873624

[20] Hossain SM Hnat T Saleheen N Nasrin NJ Noor J Ho J . mCerebrum: a mobile sensing software platform for development and validation of digital biomarkers and interventions. Proc Int Conf Embed Netw Sens Syst. (2017) 2017. 10.1145/3131672.3131694 PMC6168216 30288504

[21] Bot BM Suver C Neto EC Kellen M Klein A Bare C . The mPower study, Parkinson disease mobile data collected using researchkit. Sci Data. (2016) 3:160011. 10.1038/sdata.2016.11 26938265 PMC4776701

[22] Halchenko YO Meyer K Poldrack B Solanky DS Wagner AS Gors J . DataLad: distributed system for joint management of code, data, and their relationship. J Open Sour Softw. (2021) 6:3262. 10.21105/joss.03262 PMC11514317 39469147

[23] Littman ML. Activity recognition from accelerometer data. In Proceedings of the Seventeenth Conference on Innovative Applications of Artificial Intelligence. (2005). p. 1541–6.

[24] Wan S Qi L Xu X Tong C Gu Z. Deep learning models for real-time human activity recognition with smartphones. Mobile Netw Appl. (2020) 25:743–55. 10.1007/s11036-019-01445-x

[25] Cho Y-S Jang S-H Cho J-S Kim M-J Lee HD Lee SY . Evaluation of validity and reliability of inertial measurement unit-based gait analysis systems. Ann Rehabil Med. (2018) 42:872–83. 10.5535/arm.2018.42.6.872 30613081 PMC6325313

[26] Mundt M Koeppe A David S Witter T Bamer F Potthast W . Estimation of gait mechanics based on simulated and measured IMU data using an artificial neural network. Front Bioeng Biotechnol. (2020) 8:41. 10.3389/fbioe.2020.00041 32117923 PMC7013109

[27] Schlachetzki JCM Barth J Marxreiter F Gossler J Kohl Z Reinfelder S . Wearable sensors objectively measure gait parameters in Parkinson's disease. PLoS ONE. (2017) 12:e0183989. 10.1371/journal.pone.0183989 29020012 PMC5636070

[28] Moore ST Yungher DA Morris TR Dilda V MacDougall HG Shine JM . Autonomous identification of freezing of gait in Parkinson's disease from lower-body segmental accelerometry. J Neuroeng Rehabil. (2013) 10:19. 10.1186/1743-0003-10-19 23405951 PMC3598888

[29] Rodríguez-Martín D Samà A Pérez-López C Català A Cabestany J. Posture transition analysis with barometers: contribution to accelerometer-based algorithms. Neural Comput Applic. (2020) 32:335–49. 10.1007/s00521-018-3759-8

[30] Wang X Vouk N Heaukulani C Buddhika T Martanto W Lee J . HOPES: an integrative digital phenotyping platform for data collection, monitoring, machine learning. J Med Internet Res. (2021) 23:e23984. 10.2196/23984 33720028 PMC8074871

