# Corrigendum: JTrack: A Digital Biomarker Platform for Remote Monitoring of Daily-Life Behaviour in Health and Disease

**DOI:** 10.3389/fpubh.2022.893531

**Published:** 2022-04-11

**Authors:** Mehran Sahandi Far, Michael Stolz, Jona M. Fischer, Simon B. Eickhoff, Juergen Dukart

**Affiliations:** ^1^Research Centre Jülich, Institute of Neuroscience and Medicine, Brain and Behaviour (INM-7), Jülich, Germany; ^2^Medical Faculty, Institute of Systems Neuroscience, Heinrich Heine University Düsseldorf, Düsseldorf, Germany

**Keywords:** mobile toolkit, mobile sensing, remote monitoring, health science, biomarkers

In the original article, there was an error. The data availability statement is missing the link to the software presented in the manuscript. The accepted version had the correct statement but it was mistakenly removed by the production team.

The following correction has been made to Data Availability Statement:

For the most updated and previous versions please visit the public repository accessible at https://github.com/Biomarker-Development-at-INM7. The raw data supporting the conclusions of this article will be made available by the authors, without undue reservation.

The authors apologize for this error and state that this does not change the scientific conclusions of the article in any way. The original article has been updated.

## Publisher's Note

All claims expressed in this article are solely those of the authors and do not necessarily represent those of their affiliated organizations, or those of the publisher, the editors and the reviewers. Any product that may be evaluated in this article, or claim that may be made by its manufacturer, is not guaranteed or endorsed by the publisher.

